# Dissecting the biological relationship between TCGA miRNA and mRNA sequencing data using *MMiRNA-Viewer*

**DOI:** 10.1186/s12859-016-1219-y

**Published:** 2016-10-06

**Authors:** Yongsheng Bai, Lizhong Ding, Steve Baker, Jenny M. Bai, Ethan Rath, Feng Jiang, Jianghong Wu, Hui Jiang, Gary Stuart

**Affiliations:** 1 Department of Biology, Indiana State University, Terre Haute, IN 47809 USA; 2 The Center for Genomic Advocacy, Indiana State University, Terre Haute, IN 47809 USA; 3 Department of Mathematics and Computer Science, Indiana State University, Terre Haute, IN 47809 USA; 4 Terre Haute South Vigo High School, Terre Haute, IN 47809 USA; 5 Animal Husbandry Institute, Inner Mongolia Academy of Agricultural & Animal Husbandry Sciences, Hohhot, 010031 People’s Republic of China; 6 Department of Biostatistics, University of Michigan, Ann Arbor, MI 48109 USA

**Keywords:** TCGA, miRNA, mRNA, Cancer, Correlation, Expression, Regulation, MMiRNA-Viewer, Visualization

## Abstract

**Background:**

MicroRNAs (miRNA) are short nucleotides that interact with their target genes through 3′ untranslated regions (UTRs). The Cancer Genome Atlas (TCGA) harbors an increasing amount of cancer genome data for both tumor and normal samples. However, there are few visualization tools focusing on concurrently displaying important relationships and attributes between miRNAs and mRNAs of both cancer tumor and normal samples. Moreover, a deep investigation of miRNA-mRNA target and biological relationships across multiple cancer types by integrating web-based analysis has not been thoroughly conducted.

**Results:**

We developed an interactive visualization tool called *MMiRNA-Viewer* that can concurrently present the co-relationships of expression between miRNA-mRNA pairs of both tumor and normal samples into a single graph. The input file of *MMiRNA-Viewer* contains the expression information including fold changes between normal and tumor samples for mRNAs and miRNAs, the correlation between mRNA and miRNA, and the predicted target relationship by a number of databases. Users can also load their own input data into *MMiRNA-Viewer* and visualize and compare detailed information about cancer-related gene expression changes, and also changes in the expression of transcription-regulating miRNAs.

To validate the *MMiRNA-Viewer*, eight types of TCGA cancer datasets with both normal and control samples were selected in this study and three filter steps were applied subsequently. We performed Gene Ontology (GO) analysis for genes available in final selected 238 pairs and also for genes in the top 5 % (95 percentile) for each of eight cancer types to report a significant number of genes involved in various biological functions and pathways. We also calculated various centrality measurement matrices for the largest connected component(s) in each of eight cancers and reported top genes and miRNAs with high centrality measurements.

**Conclusions:**

With its user-friendly interface, dynamic visualization and advanced queries, we also believe *MMiRNA-Viewer* offers an intuitive approach for visualizing and elucidating co-relationships between miRNAs and mRNAs of both tumor and normal samples. We suggest that miRNA and mRNA pairs with opposite fold changes of their expression and with inverted correlation values between tumor and normal samples might be most relevant for explaining the decoupling of mRNAs and their targeting miRNAs in tumor samples for certain cancer types.

**Electronic supplementary material:**

The online version of this article (doi:10.1186/s12859-016-1219-y) contains supplementary material, which is available to authorized users.

## Background

Cancer is a disease caused by an uncontrolled division of abnormal cells, and it can start from anywhere in the human body and spread into surrounding tissues/organs. There are more than 100 types of cancer reported (http://www.cancer.gov/about-cancer/what-is-cancer). Cancer is affecting millions of lives across the world. According to the World Cancer Report, cancer rates could further increase by 50 % to 15 million new cases in the year 2020 (http://www.who.int/mediacentre/news/releases/2003/pr27/en/). The leading causes of cancer death among men are different from the leading causes of cancer death among women. Cancer occurrence also has racial/ethnic and geographic variations (http://www.cdc.gov/cancer/dcpc/data/).

The advent of next-generation sequencing (NGS) has opened a new avenue by which, in theory, all of the limitations of traditional technology can be overcome at a reasonable cost and the underlying chromosomal structure of an individual’s DNA can be fully characterized down to the nucleotide level. Next-generation sequencing technology in cancer studies has become an effective way to provide high sensitivity and resolution in the post-genomic era.

In 2006, the Cancer Genome Atlas (TCGA), a project initiated by the National Cancer Institute (NCI) and National Human Genome Research Institute (NHGRI), aimed to catalogue mutations responsible for cancer. So far, more than 30 cancer types and 10 cancer tissues (Breast, Central Nervous System, Endocrine, Gastrointestinal, Gynecologic, Head and Neck, Hematologic, Skin, Soft Tissue, Thoracic, Urologic) have been presented for potential characterization and their DNA and RNA sequencing data are publicly accessible to the community and researchers (http://cancergenome.nih.gov). The Cancer Genome Atlas project provides various types of NGS sequencing data including Exome, SNP, Methylation, mRNA, miRNA, and Clinical. Moreover, sequencing data for multiple individuals of both tumor and normal samples are available for each of these cancer types [1].

MicroRNAs (miRNA) are short nucleotides that interact with their target genes through 3′ untranslated regions (UTRs). MicroRNAs can at the same time target many mRNAs and fine-tune gene expression by means of cooperative or combinatorial targeting. In other words, one miRNA can at the same time target many RNAs, and many miRNAs can cooperatively target a single mRNA [2]. In mammals, the main effects of the miRNA-mRNA interaction are the destabilization of the target mRNAs by the pairing miRNAs [3].

It is common to attempt to describe and/or visualize miRNA-mRNA interaction networks in order to better understand their contributions to various diseases states, including cancer. Generic studies that involve normal negative correlation have been noticed. In fact, there exist more complex issues ““decoupling” like genes being either tumor promoters or suppressors depending on the tumor-type. The miRNA and mRNA pairs can fail to show negative correlation when expected, and conversion from negative to positive correlation. Specifically, a study on miRNA-mRNA network in brain regions of human alcoholics suggested that miRNAs would finally overcome the adaptive upregulation of the targeted genes and resultantly turn those initially upregulated alcohol-responsive genes to be downregulated [2]. The *hsa-miR-183* was found to be expressed higher in most of the breast cancers, but lower expressed in estrogen receptor-positive breast tumors, which suggests the *hsa-miR-183* plays different roles in different cancer cells [4]. In a study of gastric cancer, *hsa-miR-183* acts as both a tumor promoter (onco-miRNA) and tumor suppressor miRNA, which depends on the type and/or subtype of cancer [5].

Furthermore, some pairs of predicted/verified miRNA and their target mRNA were found to fail to show the anti-correlation in vivo [6]. The potential mechanisms by which a target mRNA might “avoid” or become “uncoupled” from its targeting miRNA was also explained [7]. The study across multiple TCGA cancer types by combining all cancers into a global analysis was performed [8].

We have previously developed a web interface tool *MMiRNA-Tar* [9] (http://bioinf1.indstate.edu/MMiRNA-Tar) that can calculate and plot the correlation of expression for mRNA-miRNA pairs across samples or over a time course using a pre-defined correlation cutoff and prediction confidence. *MMiRNA-Tar* provides researchers a convenient tool to calculate the co-relationship between mRNAs and miRNAs to predict their targeting relationship. In order to facilitate effective interpretation of the important attributes and values identified for each miRNA and mRNA pair, in this study we developed a prototype of the web interface tool *MMiRNA-Viewer* that can concurrently render the co-relationships of mRNA − miRNA pairs in both tumor and normal samples. We also investigated the target relationship between mRNA and miRNA pairs for TCGA cancer datasets and studied their biological functions in a systematic way.

## Methods

### Overview

**Fig. 1:**
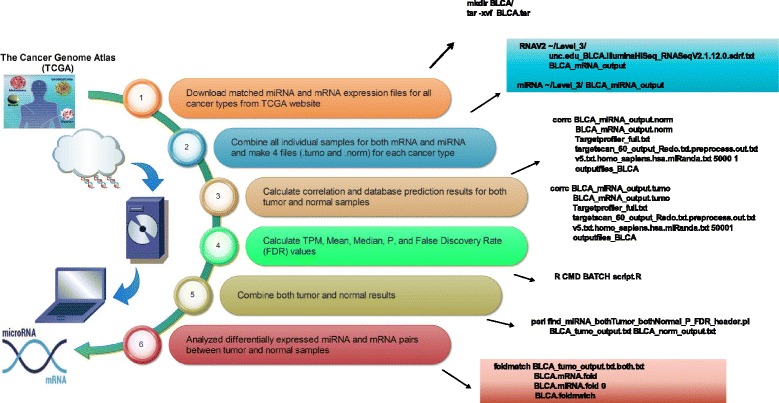
The workflow of identifying and selecting both tumor and normal pairs for eight cancer types

The workflow of identifying and selecting both tumor and normal pairs for eight cancer types is illustrated in Fig. 1. The details of each step are described below:

### Download the matched miRNA and mRNA sequencing datasets of both tumor and normal samples for available cancer types from TCGA website

We first downloaded the miRNA and mRNA expression files for all 34 cancer types from TCGA website. The expression results were taken from the TCGA Data Level 3. Specifically, miRNA-Seq data were generated by Baylor College Human Genome Sequencing Center (BCGSC), and RNA-Seq data were generated by University of North Carolina at Chapel Hill (UNC). To make measurement units between two sequencing data sets consistent, we adopted transcripts per million (TPM) expression values for both miRNA and mRNA analyses.

### Combine all individual samples for both miRNA and mRNA data for each cancer type

Every sample downloaded from TCGA contains mRNA and miRNA expression values for individual samples. We used in-house developed C programs to match patients’ tumor and normal samples in the same sample order to generate four tabular data files (tumor and normal each for mRNA and miRNA expression profiles) for each cancer type.

### Calculate correlation values and database prediction outcomes between miRNA and mRNA pairs

A customized C program was written to calculate Pearson correlation coefficient (PCC) and check three target prediction databases (TargetProfiler [10], TargetScan [11], and miRanda [12]) for prediction results of both tumor and norm samples. When we searched for the match between pre-miRNA from TCGA and mature miRNA from target prediction database, we ignored the case and omitted the last digit (tail). Although miRNA IDs with different last digits represent the distinct precursor sequences, they express identical mature sequence. A match was also called for compared cases with different lettered suffixes since they denote closely related mature sequence. The existence of the targeting relationship was claimed if a target prediction outcome was supported by at least one of the three databases mentioned above.

### Calculate statistical significance of miRNA and mRNA correlation pairs

A customized R script was written to perform normalization and calculate Transcript Per Million (TPM), Average, Median, *P*-values and False Discovery Rate (FDR) values, including multiple testing for miRNA and mRNA expression files in both tumor and norm samples. The TPM was calculated for normalized expression. The average expression was used for calculating the Fold Change (FC) between tumor and normal samples. The calculated *P*-values and FDR values were used to select statistically significant pairs.

### Select cancer types and obtain input miRNA and mRNA pairs

Among 34 cancer types, 11 cancer types do not have their normal samples; One cancer type (GBM) has no tumor sample available; The mRNA and miRNA files in tumor samples for 7 cancer types do not match their counts. Two cancer types (STAD and COAD) do not match their mRNA and miRNA counts in both tumor and normal samples. There are two cancer types (PAAD and CHOL) having no common target pairs while selecting opposite expression pairs. Two cancer types (PCPG and CESC) were excluded due to the FDR cutoff values (<0.1) that we employed. The cancer type (THYM) having only two matched tumor or normal samples was also excluded from the subsequent analysis. Therefore, we obtained eight total cancer types (UCEC, KICH, HNSC, THCA, KIRP, LIHC, LUSC, BLCA) with complete transcriptome data available for both tumor and normal samples. 29 ambiguous (“?”) genes were excluded because of a similar reason mentioned in the study [9].

### Combine both tumor and normal samples and analyze differentially expressed mRNAs and miRNAs pairs between tumor and normal samples

We combined both tumor and normal results by selecting miRNA and mRNA pairs with opposite correlation values and FDR < 0.1 and predicted by at least one of three prediction algorithms. The FC in terms of expression ratio of tumor over normal was calculated for miRNAs and mRNAs.

### Distinguish the proportion of upregulated, downregulated, and unchanged miRNAs and mRNAs between tumor and normal samples

In order to compare the regulation directional change for miRNA and mRNA between tumor and normal samples within and between cancers, The fraction of upregulated, downregulated, and unchanged for miRNAs and mRNAs were calculated. The scatter plots of the miRNAs in tumor *vs* in normal and of the mRNAs in tumor *vs* in normal for eight cancer types were plotted using a customized R script.

### Determine the importance of mRNAs and miRNAs in cancer networks

We used various centrality measures (spanning tree, degree, closeness, betweenness) to identify important mRNAs and miRNAs in tumor and normal samples for each of the eight cancers. For degree, closeness, and betweenness calculation, we used methods described in the paper [13] and implemented with tnet package (http://toreopsahl.com/tnet). The calculation of spanning tree was done using a modified method described in the paper [13] and the tools used in implementation were the octave package for matrix calculation (https://www.gnu.org/software/octave/).

We first processed all pairs selected for each cancer type. The connected components (groups) were created according to the available connection after removing duplicated nodes (mRNAs/miRNAs). We only studied the largest connected component in the analysis. Specifically, for each studied large connected component, edge lists were generated and tnet package were executed to calculate degree, closeness, and betweenness centrality. A customized script was designed to generate adjacency matrices and calculate Laplacian matrices and determinant of reduced Laplacian matrices. The centrality of spanning tree was calculated using the overall determinant and the values from Laplacian matrices. The implementations for the measurement of all centralities were done using R and customized C programs. All measurement results were normalized using the max values in its group. “Nodes” with positive values for most of centrality measurement metrics for each of eight cancers were selected for cancer annotation.

### Annotate cancer association annotation for genes and miRNAs

We annotated cancer association for gene and miRNA pairs via querying different databases. Specifically, all the genes in the table were queried to get annotations of pathways using KEGG Mapper – Search Pathway [14], of gene ontology using Ensembl BioMart [15], of cancer-related diseases using Catalogue of Somatic Mutations In Cancer (COSMIC) [16], and of human-related diseases using Online Mendelian Inheritance In Man (OMIM) [17]. All the miRNAs were queried to get the experimentally validated miRNAs and annotations of their related diseases using DIANA-TarBase [18], Human MicroRNA Disease Database (HMDD) [19], and miR2Disease [20].

Specifically, in the KEGG Mapper – Search Pathway [14], the genes in the pairs were entered as objects to search against ko with all the default parameters. In the Esembl Biomart [15], the genes in the pairs were put into the input external references with an ID list of HGNC symbol(s) to search against the Ensembl Genes 84 Homo sapiens genes (GRCh38.p5) with all the default parameters. From the COSMIC website [16], the Cancer Gene Census file was downloaded and parsed. From the OMIM website [17], the genemap2.txt file was downloaded and parsed. On the Diana tools TarBase website [18], each miRNA was searched to get its related diseases. From the HMDD website [19], the whole dataset of miRNA-disease association data file was downloaded and parsed. On the miR2Disease website [20], the “All Entries” file was downloaded and parsed.

The annotations of genes and experimentally validated miRNAs from the above-mentioned databases were manually scrutinized, categorized, and tagged to tell whether they were related to the eight cancer types in this study. The annotations that were not related to the eight cancer types were not included. After the annotation filtering step, each cancer type that the gene and miRNA pair belongs to were classified into three categories: the cancer type was found in neither gene nor miRNA annotations(neither_exist), in either gene or miRNA annotations (either_exist), and in both gene and miRNA annotations (both_exist).

### Analyze gene functional enrichment for eight cancers

We searched the Database for Annotation, Visualization and Integrated Discovery (DAVID) [21] for functional information about smaller sets of genes with their predicted targeting miRNAs having high correlation values and reported clusters with enrichment scores greater than 0.99. We also did ClueGo of Cytoscape plug-in [22] functional annotation for larger sets of genes which were selected according to the criteria that pairs’ correlation values to the top 5 % (95 percentile) in either tumor or normal samples in each cancer type due to the fact that the range of correlation values varies substantially among different cancer datasets.

We also ran ClueGo for a bigger size of gene list. This set of genes was selected according to the criteria that pairs’ correlation values to the top 5 % (95 percentile) in either tumor or normal samples in each cancer type due to the fact that the range of correlation values varies substantially among different cancer datasets.

### Visualize relationships between miRNA and mRNA pairs using *MMiRNA-Viewer*

Only pairs with high confidence (FDR values are less than 0.1) were uploaded into *MMiRNA-Viewer* for visualization. The visualization graph presented by *MMiRNA-Viewer* is supported by the Node JS Application (D3.js and JQuery: https//d3js.org) with browser side application prototype. The algorithm for drawing out the graph starts with the links between mRNA and miRNA pairs. Links indicate the databases that validated the connection and normal/tumor correlation. We use link color intensity to represent the number of predictions by different databases. Then nodes are drawn based on its type. Basically we use squares to represent mRNA while circles represent miRNA. Motion actions like click, drag, and double click are also attached to the nodes.

The *MMiRNA-Viewer* can be played using the following steps:

1. Upload input miRNA and mRNA pair data. The file uploaded to the this tool is a text file and should contain expression correlations between mRNA and microRNA, mRNA and microRNA normal/tumor P() and FDR values, Number of Databases that validated the connection and Normal/Tumor correlation, and Fold Changes values for miRNA and mRNA pairs.
2. Search and filter miRNA and mRNA pairs. Users can search for a specific node in the graph by inserting the gene name in the search box. After inputting an mRNA or microRNA name, the user can click the search button to center its position in the graph. The ID search is case-sensitive. Additionally, there are various filters that can be applied to filter data at will. For example, when users select Node Filter as “mRNA” and Connections Filter as “>10”, then only the nodes representing mRNA with connected nodes greater than 10 will be highlighted in the graph.
3. Show miRNA and mRNA pair data. Users can click on two nodes that are connected with each other to get the annotation values in normal and tumor samples, which are directly displayed in the table right below the “Filters”. Users can collapse and expand the legend on the top right corner in the graph by clicking the legend icon.

### Calculate Pearson Correlation Coefficient (PCC) values between *MMiRNA-Viewer* and *CrossHub* for 100 miRNA and mRNA pairs in the HNSC tumor data set

To demonstrate merits of the co-relationship for mRNA-miRNA calculated by *MMiRNA-Viewer,* we compared our *MMiRNA-Viewer* with CrossHub [23], a tool that can also use the TCGA mRNA RNA-seq data and miRNA-seq data to calculate the expression correlations between miRNAs and mRNAs of each cancer type. To do so, we selected 100 unique genes and miRNAs from calculated HNSC_tumor_output data set, which contains 15,870 genes and 398 miRNAs, The shell script “miRNA-Seq-RNA-Seq.co-expr.sh” in CrossHub.1.3.3 was used to calculate the PCC values.

## Results

### miRNA and mRNA data samples

The number of cancer and normal samples downloaded from TCGA for each cancer type are listed in Table 1.

**Table 1:** The number of cancer and normal samples downloaded from TCGA

Cancer types	Number of normal samples	Number of cancer samples	Total
ACC	0	79	79
**BLCA**	**11**	**53**	**64**
BRCA	87	776	863
CESC	3	306	309
CHOL	9	36	45
COAD	8	223	231
DBLC	0	47	47
ESCA	11	186	197
FPPP	0	45	45
GBM	5	0	5
**HNSC**	**44**	**480**	**524**
**KICH**	**25**	**66**	**91**
KIRC	71	261	332
**KIRP**	**32**	**291**	**323**
LAML	0	173	173
LGG	0	530	530
**LIHC**	**50**	**370**	**420**
LUAD	20	455	475
**LUSC**	**38**	**342**	**380**
MESO	0	87	87
OV	0	299	299
PAAD	4	179	183
PCPG	3	184	187
PRAD	52	498	550
READ	3	91	94
SARC	0	261	261
SKCM	1	450	451
STAD	37	377	414
TGCT	0	156	156
**THCA**	**59**	**512**	**571**
THYM	2	120	122
**UCEC**	**13**	**174**	**187**
UCS	0	57	57
UVM	0	80	80

*Notes*: The bold rows are cancer types selected for the analysis

### Significant and inversely correlated miRNA and mRNA pairs

**Fig. 2:**
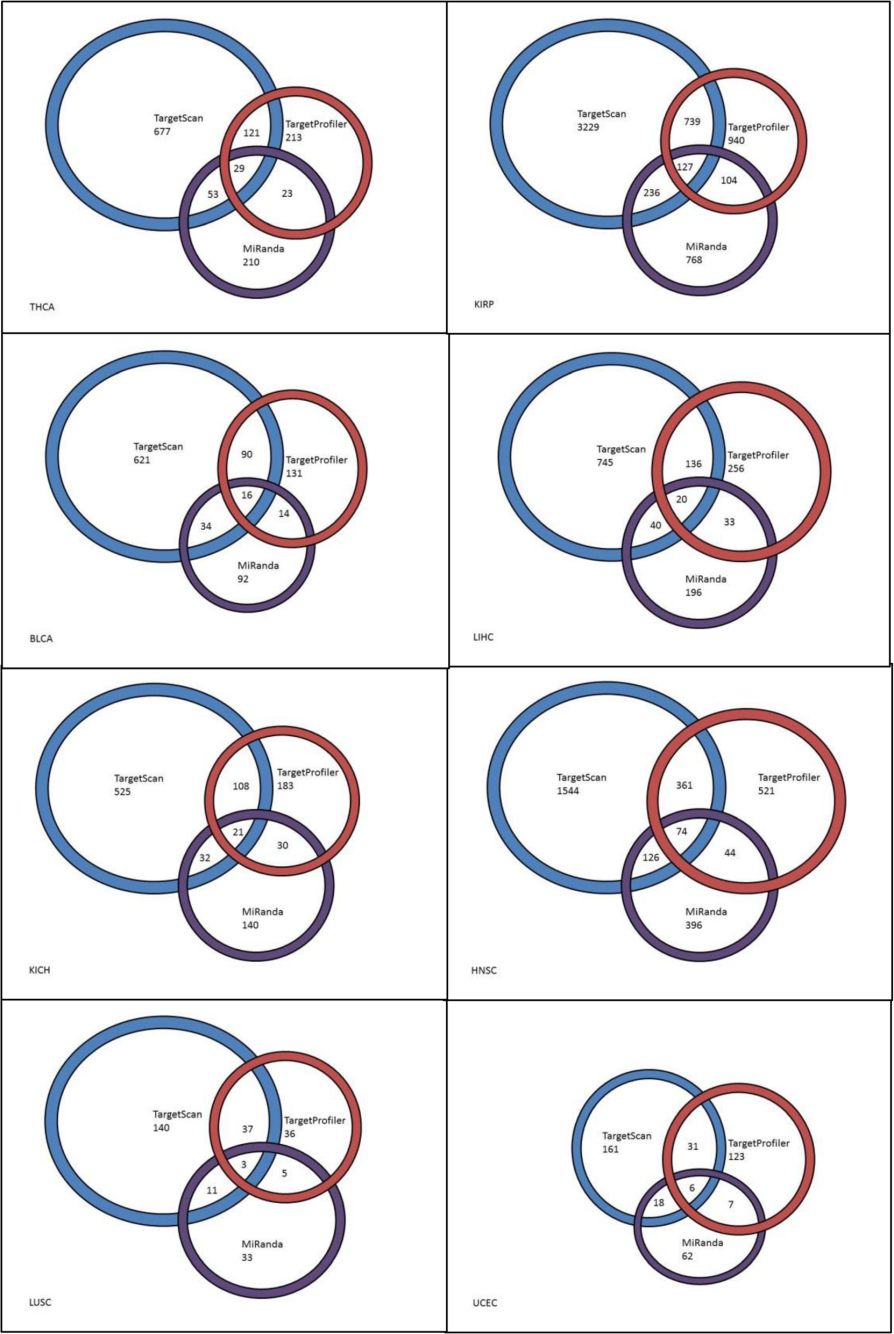
Graphical representations of the predicted miRNA & mRNA relationships by TargetProfiler, TargetScan, and miRanda software. All relationships shown are mutually exclusive from other groupings. Sizes of rings are relative to the amount of relationships detected by each software tool

Upon excluding cancer types that do not have matched miRNA and mRNA samples and running customized expression calculation and database prediction scripts, the filtered expression data for the eight selected cancer types are generated. We obtained 14,505 total miRNA and mRNA pairs (both tumor and normal types) which met the statistical cutoff criteria (inverse correlation values and FDR < 0.1) for eight cancer types (Additional file 1). The target prediction results by three selected databases are shown in Fig. 2. It can be clearly seen that TargetScan consistently predicts more targeting pairs than the two other tools. The result indicates that the target prediction algorithms show similar prediction trends as in our previous study [9].

### Opposite FC miRNA and mRNA pairs

Among 14,505 pairs, we identified 238 pairs meeting the condition of opposite FC between tumor and normal samples (Additional file 2). These pairs could be cases where mRNAs are regulated by miRNAs in opposite ways. In addition, we found that KICH, KIRP, and HNSC contain the most number of mRNA-miRNA pairs with opposite FC values between tumor and normal samples. On the other hand, LUSC is the cancer with the least number of pairs with opposite FC values between tumor and normal samples. We did not find any significant pairs with mRNA up-regulation (FC > 1) and miRNA down-regulation (FC < 1) in UCEC (Additional file 3).

### Common miRNA and mRNA pairs across cancers

We also compared the number of common mRNA-miRNA pairs across the eight cancer types. We observed that the overlapping pairs between cancers are not consistent (Table 2). In particular, KIRP and HNSC have the highest overlap pairs without counting KIRP and KICH because both are kidney cancer related.

**Table 2:** The number of common mRNA-miRNA pairs across 8 cancer types

	KIRP	LIHC	UCEC	KICH	HNSC	THCA	BLCA	LUSC
KIRP	-	31	10	49	41	28	6	2
LIHC	31	-	3	3	12	3	1	3
UCEC	10	3	-	0	2	3	3	0
KICH	49	3	0	-	7	4	2	0
HNSC	41	12	2	7	-	11	6	1
THCA	28	3	3	4	11	-	5	1
BLCA	6	1	3	2	6	5	-	0
LUSC	2	3	0	0	1	1	0	-

### Distribution of upregulated, downregulated, and unchanged miRNAs and mRNAs between tumor and normal

**Fig. 3:**
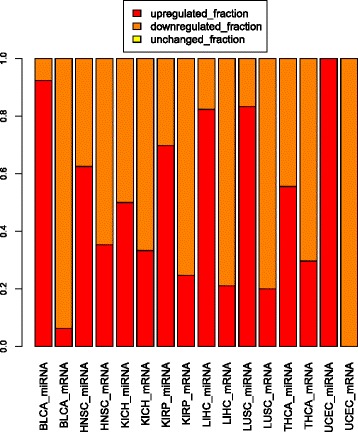
The distribution of upregulated, downregulated and unchanged miRNAs and mRNAs in 238 pairs

In different cancers the ratio of upregulated, downregulated and unchanged miRNAs and mRNAs are different among cancers (Fig. 3). Especially, the high fraction of upregulation of miRNAs are constantly accompanied by the high fraction of downregulation of the mRNAs. The extreme case is the UCEC, which has all the miRNA upregulated and all the mRNA downregulated. There are relatively higher percentages of miRNAs upregulated in each of the BLCA, HNSC, KIRP, LIHC, LUSC, and UCEC, whereas there are fewer percentages of miRNAs upregulated in the KICH and THCA. The scatter plot of differentially expressed genes in miRNA and mRNA for all 238 pairs in eight cancers is shown in Additional file 4.

### Connections in significant miRNA and mRNA correlation pair for eight cancers

**Fig. 4:**
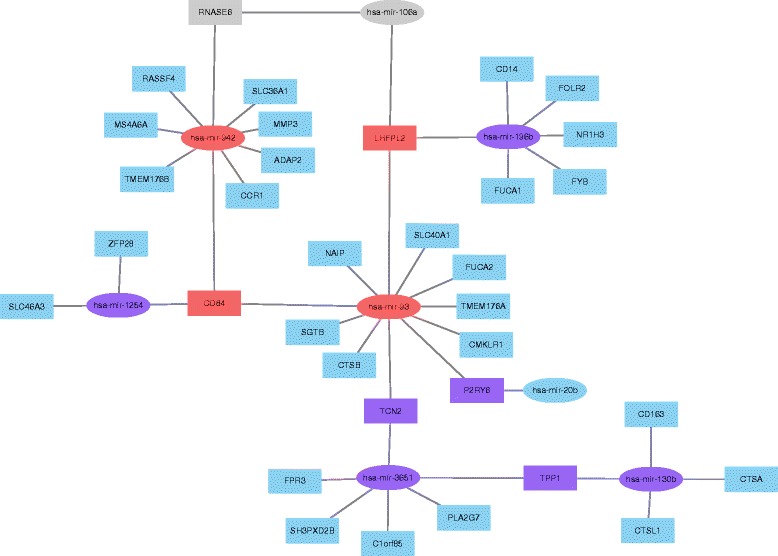
The connection topology for one of the top clusters with 42 “nodes” in LUSC

We obtained connection groups for each selected cancer and calculated various centrality matrices as mentioned in the Method section. All selected cancers except LUSC contain only one connected group with greater than 20 connections/nodes (LUSC: 71; BLCA: 810; HNSC: 2954; KICH: 926; KIRP: 6109; LIHC: 1264; THCA: 1164; UCEC: 193). The nodes are selected genes and miRNAs in the largest group for each cancer. In LUSC’s case, there are three groups with node size greater than 20. A connection topology for one of the top clusters with 42 “nodes” in LUSC is shown in Fig. 4.

The centrality scores of genes and miRNAs based on various matrix measurement for the largest connected component of eight cancer types were calculated and shown in Additional file 5.

### Cancer association annotation for genes and miRNAs with positive centrality measurement values

When looking at the gene and miRNA cancer association, the results revealed one hit of “both_exist”, 135 hits of “either_exist”, and 102 hits of “neither_exist”. The both_exist gene and miRNA pair is fibroblast growth factor receptor 3 (*FGFR3)* and *hsa-miR-100* in the BLCA. *FGFR3* and *hsa-miR-100* pair in BLCA have been identified by this approach and both of them were reported as the bladder cancer gene and miRNA. In other words, in the BLCA cancer dataset, the top negatively correlated gene and miRNA in expression values were both annotated to be related to the cancer BLCA in the above-mentioned databases. Specifically, *FGFR3* gene is reported to be involved in Bladder cancer based on the result (hsa05219) from the KEGG pathway mapping database [14]. Previous studies [24] showed that the loss of *hsa-miR-100* leads to upregulation of *FGFR3* before its mutation. Therefore, literature provide the support that our bioinformatics pipeline can detect such pairs with the opposite regulation between mRNAs and miRNAs. Literature search results associated with cancers for other pairs which are reported here to have opposite FC values between mRNAs and miRNAs and inverse correlations between tumor and normal samples are shown in Additional file 6.

In the nodes of eight cancer types, there are 510 nodes (Genes/miRNAs) across cancer types, namely appearing in more than two different cancer types. There is one gene node across four cancer types.

There are 3 miRNA nodes across 7 cancer types. The *hsa-miR-203* was decreased in esophageal adenocarcinoma compared to paired adjacent mucosa [25]; the *hsa-miR-221* was downregulated in prostate cancer [26]; *hsa-miR-222* was upregulated in anaplastic thyroid carcinoma [27]. There are 4 miRNA nodes across 6 cancer types. The *hsa-miR-182* was upregulated in the esophageal cancer [28]. The *hsa-miR-183* was upregulated in the metastatic breast cancer [29]. The *hsa-miR-21* was upregulated in the gastric cancer [30]. The *hsa-miR-629* that was functionally inhibited could suppress motility and invasion of the clear cell renal cell carcinoma [31]. There are 9 miRNA nodes across 5 cancer types. The *hsa-miR-106b* was downregulated in the metastatic pancreatic cancer [32]. The *hsa-miR-1266* was significantly decreased in expression in gastric cancer tissues [33]. The has-mir-141 could downregulate the expression of TM4SF1 to inhibit the pancreatic cancer cells’ migration and invasion [34]. The *hsa-miR-145* was downregulated in laryngeal squamous cell carcinoma [35]. The *hsa-miR-378* was downregulated in cutaneous squamous cell carcinoma [36]. The *hsa-miR-454* was downregulated in osteosarcoma [37]. The *hsa-miR-484* was upregulated in expression in the serum samples of early breast cancer patients [38]. The *hsa-miR-625* was upregulated in bile duct cancer [39]. The *hsa-miR-940* was downregulated in hepatocellular carcinoma tissues and cell lines [40].

### Gene functional enrichment analysis

We searched the Database for Annotation, Visualization, and Integrated Discovery (DAVID) [21] for functional information about 238 pairs of genes with their predicted targeting miRNAs at high correlation values. Enrichment of these genes was found in several GO biological processes. Some of these genes are involved in protein transport and localization, Some of these genes are involved in regulation of protein modification process, some of these genes are associated with oxidation-reduction, and some genes are involved in protein modification metabolic process. Genes associated GO terms from 238 pairs are shown in Table 3.

**Table 3:** Gene Ontology (GO) terms for 238 selected genes

Cancer Type	GO Term	Genes
KIRP,LIHC	GO:0000267 ~ cell fraction	*ABCA3,ADRA1B,DTNA,ENG,FMR1,GPR143,LRP1B,MME,PCSK1N,PDLIM5,PRKCZ,RAPGEF3,SLC26A2,UGT1A1*
HNSC,LIHC	GO:0001932 ~ regulation of protein amino acid phosphorylation	*CD3E,ENG,FGF2,PRKCZ,RAPGEF3*
KIRP,LIHC	GO:0005624 ~ membrane fraction	*ABCA3,ADRA1B,DTNA,ENG,GPR143,LRP1B,MME,PDLIM5,PRKCZ,RAPGEF3,SLC26A2,UGT1A1*
KIRP,LIHC	GO:0005626 ~ insoluble fraction	*ABCA3,ADRA1B,DTNA,ENG,GPR143,LRP1B,MME,PDLIM5,PRKCZ,RAPGEF3,SLC26A2,UGT1A1*
BLCA,KICH	GO:0005856 ~ cytoskeleton	*ABI2*,*CDC14A*,*ESPL1*,*KIF13B*,*MYO5B*,*NAV1*,*PDLIM2*,*PDLIM5*,*PKD2*,*SGCE*,*TUBE1*
BLCA,THCA	GO:0005886 ~ plasma membrane	*ABI2*,*AGPAT1*,*ANTXR1*,*FGFR3*,*NBEA*,*PARVA*,*PIK3C2A*,*SGCE*,*SVIL*
HNSC,LIHC	GO:0019220 ~ regulation of phosphate metabolic process	*ADCY4*,*CD3E*,*ENG*,*FGF2*,*PRKCZ*,*RAPGEF3*
BLCA,KICH	GO:0019899 ~ enzyme binding	*ABI2*,*ATN1*,*KIF13B*,*NBEA*,*PDLIM5,PKD2,RAD18*
HNSC,LIHC	GO:0031399 ~ regulation of protein modification process	*CD3E,ENG,FGF2,NDFIP2,PRKCZ,RAPGEF3*
HNSC,LIHC	GO:0032268 ~ regulation of cellular protein metabolic process	*CD3E,EIF2C3,ENG,FGF2,NDFIP2,PRKCZ,RAPGEF3*
HNSC,LIHC	GO:0042325 ~ regulation of phosphorylation	*ADCY4,CD3E,ENG,FGF2,PRKCZ,RAPGEF3*
BLCA,KICH	GO:0043228 ~ non-membrane-bounded organelle	*ABI2,CDC14A,ESPL1,KIF13B,MYO5B,NAV1,PDLIM2,PDLIM5,PKD2,RAD18,SGCE,TUBE1*
BLCA,KICH	GO:0043232 ~ intracellular non-membrane-bounded organelle	*ABI2,CDC14A,ESPL1,KIF13B,MYO5B,NAV1,PDLIM2,PDLIM5,PKD2,RAD18,SGCE,TUBE1*
HNSC,LIHC	GO:0051174 ~ regulation of phosphorus metabolic process	*ADCY4,CD3E,ENG,FGF2,PRKCZ,RAPGEF3*
KIRP,LIHC	GO:0055114 ~ oxidation reduction	*AKR7A2,GRHPR,HSD17B1,IVD,MOSC2,OGFOD1,RETSAT,SC5DL*

We used Cytoscape ClueGO plugin to do the GO enrichment of the 32 files of the top 5 % expression correlation coefficient of different levels at Normal High (NH), Normal Low (NL), Tumor High (TH), Tumor Low (TL) genes of eight cancers. Only BLCA, HNSC, KICH, KIRP, LIHC, and THCA have GO enrichment results. BLCA GO terms were only found in TL dataset, so we only used HNSC, KICH, KIRP, LIHC, and THCA to discover GO terms that would be shared by NH and TL or by NL and TH in each of the five cancers.

The GO terms shared by NH and TL or by NL and TH in HNSC, KICH, KIRP, LIHC, and THCA were denoted by Venn diagrams (Additional file 7). Only HNSC, KICH, and KIRP had GO terms shared by NH and TL or by NL and TH. In LIHC and THCA there was no GO terms shared by NH and TL or by NL and TH. We found that there were 35 shared GO terms in HNSC_shared_GO_in_NH_TL, 2 in HNSC_shared_GO_in_NL_TH, 41 in KICH_shared_GO_in_NH_TL, 2 in KICH_shared_GO_in_NL_TH, 99 in KIRP_shared_GO_in_NH_TL, 47 in KIRP_shared_GO_in_NL_TH, 0 in LIHC_shared_GO_in_NH_TL, 0 in LIHC_shared_GO_in_NL_TH, 0 in THCA_shared_GO_in_NH_TL, and 0 in THCA_shared_GO_in_NL_TH.

It is interesting that the HNSC_shared_GO_in_NH_TL and KICH_shared_GO_in_NH_TL have many common GO terms like mitotic sister chromatid segregation, G1/S transition of mitotic cell cycle, regulation of transcription involved in G1/S transition of mitotic cell cycle, and others. HNSC_shared_GO_in_NL_TH and KICH_shared_GO_in_NL_TH have the exact two common GO terms, histone deacetylase complex and positive regulation of transporter activity. These common GO terms suggest that the HNSC and KICH cancer have genes of similar functions that interacts with the miRNA, but the expression correlation coefficients between these genes and miRNAs are reversed by both cancers. Genes associated GO terms for these larger datasets are shown in Additional file 8.

#### MMiRNA-Viewer

**Fig. 5:**
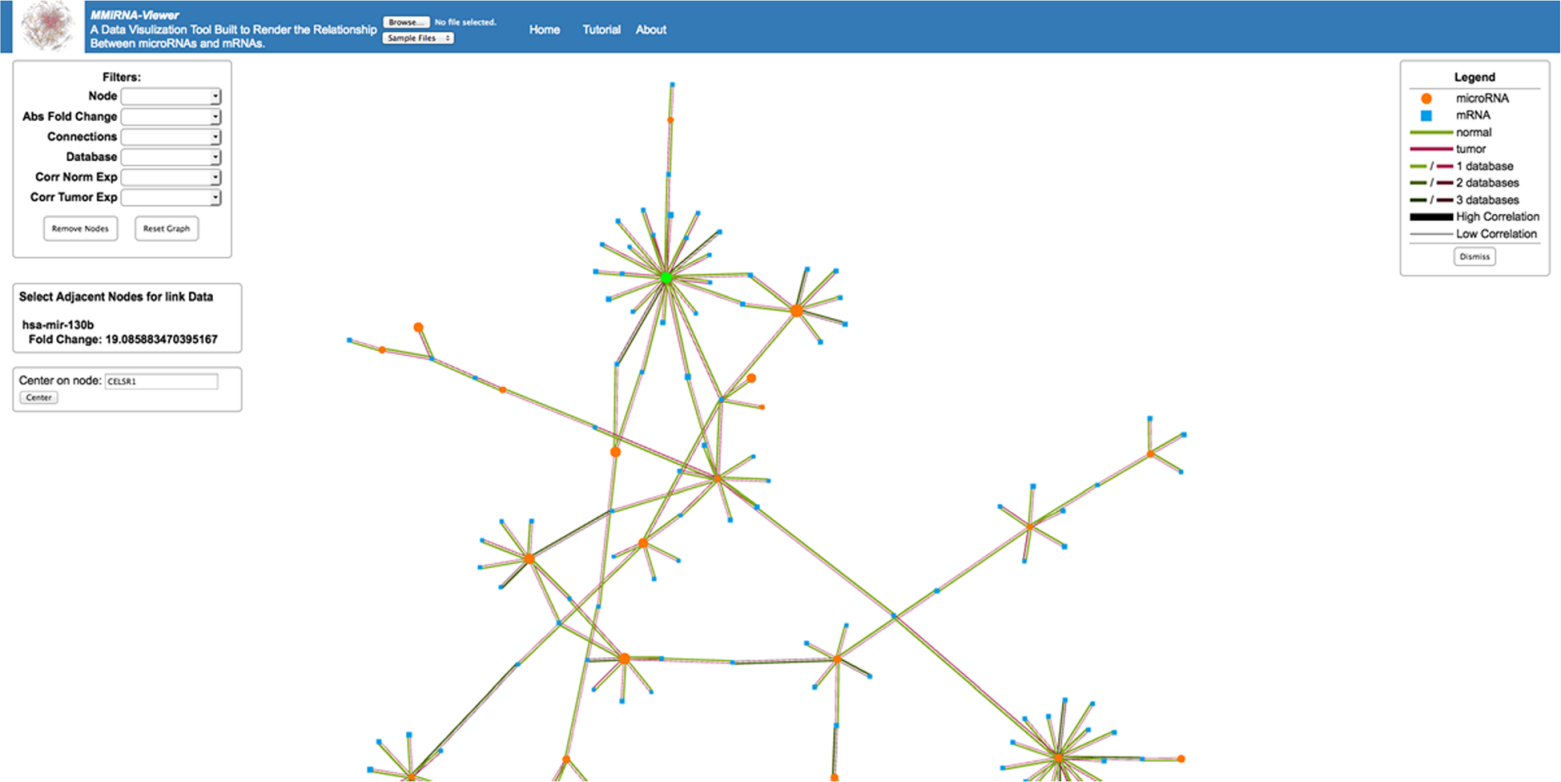
An interface screenshot of *MMiRNA-Viewer*

We have developed a prototype visualization tool -*MMiRNA-Viewer* with a friendly user interface. Currently *MMiRNA-Viewer* supports various types of miRNA-mRNA co-expression profile data. It also has the function of filtering various miRNAs and mRNA pairs for better prediction accuracy. The tool also presents analysis results in intuitive visualizations and support dynamic uploading and comparison of files to help users search biological annotation of customized miRNAs and mRNAs pairs. Specifically, the graph shows two types of nodes summarizing average expression information for mRNAs and miRNAs, and two types of links representing target relationships between miRNA and mRNA pairs in normal and tumor samples. Users can visualize detailed information about cancer-related gene expression changes, and also changes in the expression of transcription-regulating miRNAs for well-characterized cancer genomes. Users can also drag nodes and their associated partners to isolated positions for better visualization. This can help users to better study the interaction relationships of a miRNA and mRNA pair and can make a node-link diagram more suitable for publication. Moreover, with displayable gene/miRNA labeling for each node, users can capture the screenshots of some sub-networks for their publications. Not only does *MMiRNA-Viewer* allow for the viewing of a single sample’s mRNA miRNA pairs, but this tool also has the functionality to display pairs in common between two separate files (e.g. customized input file vs built-in TCGA datasets). An interface screenshot of *MMiRNA-Viewer* is shown in Fig. 5.

*MMiRNA-Viewer* is available at http://bioinf1.indstate.edu/MMiRNA-Viewer.

### Comparison of Pearson Correlation Coefficient (PCC) values between *MMiRNA-Viewer* and *CrossHub* for 100 miRNA and mRNA pairs

When compared to a short running time (less than one minute) using *MMiRNA-Viewer*, it took CrossHub 9 min to calculate correlation coefficient values for 1836 pairs. The average value of absolute difference between two methods is ~0.07. The calculated correlation values by two methods are shown in Additional file 9.

## Discussions

In this study, we developed a web-based tool *MMiRNA-Viewer* to upload input data files and visualize the results in an intuitive way that user can quickly locate their interested pairs for further functional study. The current version has many interesting features, but its functionalities can be further expanded. For instance, we would like to incorporate other functionalities with publicly available literature search and pathways mapping study.

Our study assumed that the tumor is activated via mRNAs mutation directly targeted by miRNAs. Results that miRNAs and mRNAs are inversely correlated exist in 238 selected pairs for eight studied cancers. Our protocol that inversely correlated miRNA and mRNA pairs should be oppositely regulated in tumor samples could be useful for prioritizing cancer associated pairs.

Current example input files for *MMiRNA-Viewer* were generated using TCGA cancer sequencing data (Level 3) to compute the correlation coefficients and target relationships for miRNA and mRNA pairs between tumor and normal samples using three state-of-art database prediction algorithms. We took FDR cutoff (<0.1) to select significantly correlated miRNA and mRNA pairs in the initial step so that more pairs potentially associated with cancers through inverse correlation could be included. Under the assumption that miRNA and mRNA pairs are often inversely correlated and their FC values should be opposite between tumor and normal samples, we obtained 238 pairs for GO biological function and pathway studies.

We employed a modified spanning tree centrality method for calculation. We have found that due to the definition of spanning tree, some nodes have pre-determined values of simply 1 and 0 and thus are not very interesting and can be predicted. This does help to calculate the result easier as we can remove some nodes and still generate the exact results for all nodes that cannot be predicted, thus increasing speed and precision of the calculation. We can then go back and fill in or modify the predictable values. Further, we have introduced a method to modify the value of the 1 s to better reflect their actual centrality while still using the spanning tree method as a back bone. For KIRP cluster, we used an altered approach for the calculation of spanning tree due to the large number of connections in the cluster. Specifically, the algorithm first found the most distant point from each “node” (gene or miRNA), then broke the nodes into two groups and removed branches from each two halves to calculate adjusted spanning tree values. Finally, two sets of results were merged according to original connections to report final spanning tree values.

Unlike our algorithm that filters out the pairs using *P*-values and FDR of the calculated correlation coefficients and using the above-mentioned 3-database matching, CrossHub, doesn’t filter out the calculated correlation coefficients of miRNAs and mRNAs via their reported *P*-values, via unreported FDR of their correlation coefficients, and via their reported miRNA-mRNA targeting databases. The two programs are basically consistent with respect to the calculation of the expression correlation coefficients of the miRNA-mRNA in the same dataset (HNSC) in TCGA. The slight difference of calculated correlation coefficient values might be due to the fact that CrossHub adopted read number mapped to each genes, whereas *MMiRNA-Viewer* uses TPM, so the mapping process might cause differences. In CrossHub pipeline, “By default, CrossHub performs the analysis for top 30 overexpressed microRNA with read counts (across all the samples) from 20,000 to 4,000,000. These miRNA represent the most important fraction in the context of biological regulation”. In addition, the memory usage of CrossHub is huge and running time is quite long.

## Conclusions

We developed an intuitive graph visualization tool *MMiRNA-Viewer* that provides a user interface for various cancer types of miRNA-mRNA expression data with significant interaction pairs. Our *MMiRNA-Viewer* supports dynamic queries through intuitive user interactions to help users search meaningful cancer biological findings. We also suggested that miRNA and mRNA pairs with opposite FC values of their expression and with inverted correlation values between tumor and normal samples might be potential pairs to be responsible for transcription decoupling for certain cancer types.

## Additional files

Additional file 1: 14,505 total miRNA and mRNA pairs (both tumor and normal types) which met the statistical cutoff criteria (inverse correlation values and FDR < 0.1). This file contains 14,505 pairs for eight cancer types. (XLSX 1655 kb)
Additional file 2: 238 miRNA and mRNA pairs (both tumor and normal types) which met the cutoff criteria of inverse correlation and opposite fold change values and FDR < 0.1. This file contains 14,505 pairs for eight cancer types. (XLSX 93 kb)
Additional file 3: Statistics of the presence in eight cancer types for 238 miRNA and mRNA pairs (both tumor and normal types) which met the cutoff criteria of inverse correlation and opposite fold change values and FDR < 0.1. (XLSX 48 kb)
Additional file 4: The scatter plot of differentially expressed genes in miRNA and mRNA for all 238 pairs in eight cancers. In this file, the expression mean values of the miRNA and mRNA of the 238 pairs of each cancer are plotted. The X-axis value of a dot in the plot is the miRNA or mRNA average expression value in the normal tissues. The Y-axis value of a dot in the plot is the miRNA or mRNA average expression value in the tumor tissues. (PDF 331 kb)
Additional file 5: The centrality scores of genes and miRNAs based on various matrix measurement for the largest connected component of eight cancer types. This file contains results for the largest cluster for all seven other cancers and the second largest cluster for LUSC. (XLSX 886 kb)
Additional file 6: Cancer association results for 238 pairs which are reported to have opposite FC values between mRNAs and miRNAs and inverse correlations between tumor and normal samples. (XLSX 23 kb)
Additional file 7: The Venn Diagrams of GO terms overlapping for the top 5 % expression correlation coefficient of different levels at Normal High (NH), Normal Low (NL), Tumor High (TH), Tumor Low (TL) genes of eight cancers. (PDF 920 kb)
Additional file 8: The Genes and their associated GO terms for the top 5 % expression correlation coefficient of different levels at Normal High (NH), Normal Low (NL), Tumor High (TH), Tumor Low (TL) genes of eight cancers. (XLSX 44 kb)
Additional file 9: Comparison of correlation coefficient values between MMiRNA-Viewer and CrossHub (XLSX 156 kb)

## Abbreviations

UTRs: Untranslated regions
TCGA: The Cancer Genome Atlas
GO: Gene ontology
NGS: Next-generation sequencing
NCI: National Cancer Institute
NHGRI: National Human Genome Research Institute
BCGSC: Baylor College Human Genome Sequencing Center
UNC: University of North Carolina at Chapel Hill
TPM: Transcripts Per Million
PCC: Pearson correlation coefficient
FDR: False discovery rate
FC: Fold change
KEGG: Kyoto encyclopedia of genes and genomes
COSMIC: Catalogue of somatic mutations in cancer
OMIM: Online mendelian inheritance in man
HMDD: Human MicroRNA disease database
DAVID: Database for annotation, visualization and integrated discovery
ACC: Adrenocortical carcinoma
BLCA: Bladder urothelial carcinoma
BRCA: Breast invasive carcinoma
CESC: Cervical squamous cell carcinoma and endocervical adenocarcinoma
CHOL: Cholangiocarcinoma
COAD: Colon adenocarcinoma
DLBC: Lymphoid neoplasm diffuse large B-cell Lymphoma
ESCA: Esophageal carcinoma
FPPP: FFPE Pilot Phase II
GBM: Glioblastoma multiforme
HNSC: Head and neck squamous cell carcinoma
KICH: Kidney chromophobe
KIRC: Kidney renal clear cell carcinoma
KIRP: Kidney renal papillary cell carcinoma
LAML: Acute myeloid leukemia
LGG: Brain lower grade glioma
LIHC: Liver hepatocellular carcinoma
LUAD: Lung adenocarcinoma
LUSC: Lung squamous cell carcinoma
MESO: Mesothelioma
OV: Ovarian serous cystadenocarcinoma
PAAD: Pancreatic adenocarcinoma
PCPG: Pheochromocytoma and Paraganglioma
PRAD: Prostate adenocarcinoma
READ: Rectum adenocarcinoma
SARC: Sarcoma
SKCM: Skin cutaneous melanoma
STAD: Stomach adenocarcinoma
TGCT: Testicular germ cell tumors
THCA: Thyroid carcinoma
THYM: Thymoma
UCEC: Uterine corpus endometrial carcinoma
UCS: Uterine carcinosarcoma
UVM: Uveal melanoma
