# Supplementary figures and images for: Dissecting the biological relationship between TCGA miRNA and mRNA sequencing data using MMiRNA-Viewer
Source: BMC Bioinformatics. 2016 Oct 6;17(Suppl 13):336. doi: 10.1186/s12859-016-1219-y (PMC5073992; doi:10.1186/s12859-016-1219-y)

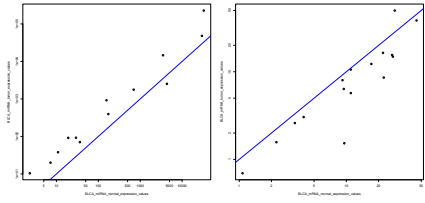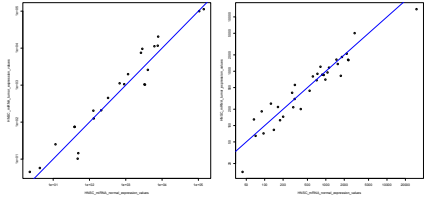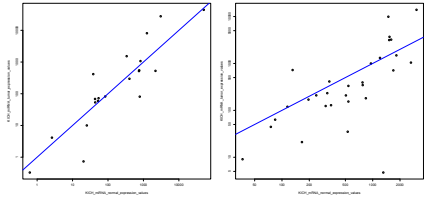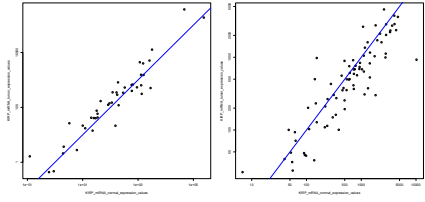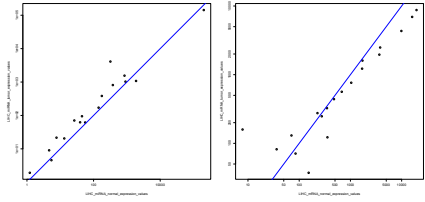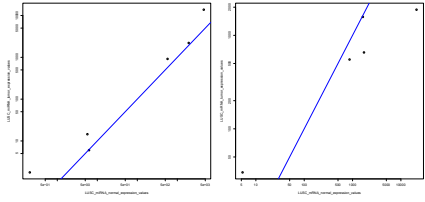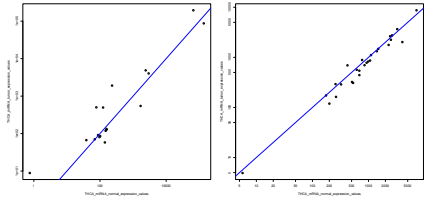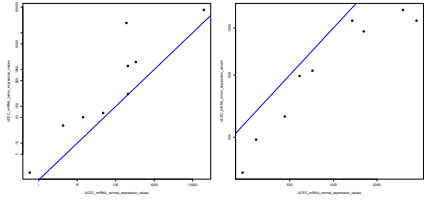

Supplement: Additional file 4: — The scatter plot of differentially expressed genes in miRNA and mRNA for all 238 pairs in eight cancers. In this file, the expression mean values of the miRNA and mRNA of the 238 pairs of each cancer are plotted. The X-axis value of a dot in the plot is the miRNA or mRNA average expression value in the normal tissues. The Y-axis value of a dot in the plot is the miRNA or mRNA average expression value in the tumor tissues. (PDF 331 kb) [file 12859_2016_1219_MOESM4_ESM.pdf]
