# Supplementary material for: Dissecting the biological relationship between TCGA miRNA and mRNA sequencing data using MMiRNA-Viewer
Source: BMC Bioinformatics. 2016 Oct 6;17(Suppl 13):336. doi: 10.1186/s12859-016-1219-y (PMC5073992; doi:10.1186/s12859-016-1219-y)

HNSC_shared_GO_in_NH_TL

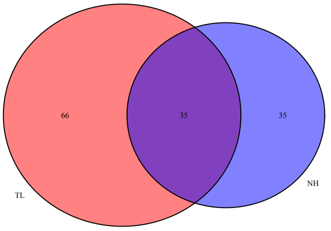

HNSC_shared_GO_in_NL_TH

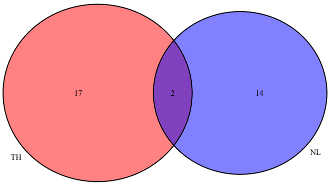

KICH_shared_GO_in_NH_TL

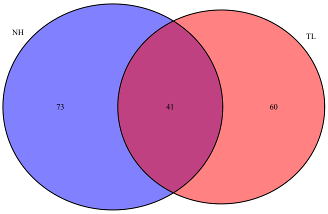

KICH_shared_GO_in_NL_TH

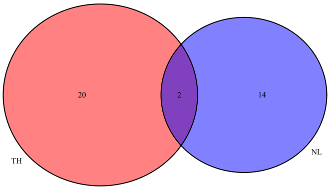

KIRP_shared_GO_in_NH_TL

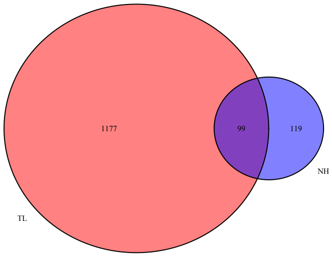

KIRP_shared_GO_in_NL_TH

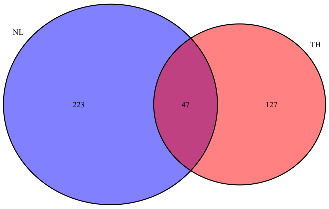

LIHC_shared_GO_in_NH_TL

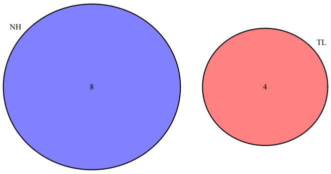

LIHC_shared_GO_in_NL_TH

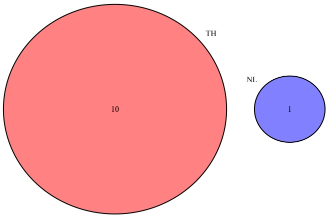

THCA_shared_GO_in_NH_TL

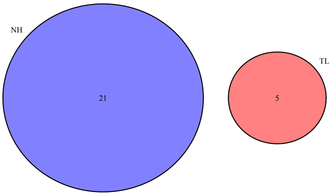

THCA_shared_GO_in_NL_TH

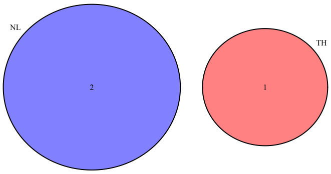

Supplement: Additional file 7: — The Venn Diagrams of GO terms overlapping for the top 5 % expression correlation coefficient of different levels at Normal High (NH), Normal Low (NL), Tumor High (TH), Tumor Low (TL) genes of eight cancers. (PDF 920 kb) [file 12859_2016_1219_MOESM7_ESM.pdf]
